# Impact of patient care teams on blood pressure control in patients with hypertension: a systematic review and meta-analysis

**DOI:** 10.1038/s41440-025-02152-9

**Published:** 2025-02-17

**Authors:** Yuichi Akasaki, Yasunori Suematsu, Kengo Azushima, Yuhei Shiga, Atsushi Sakima, Michihiro Satoh, Hisatomi Arima, Nobuhito Hirawa

**Affiliations:** 1 Department of Cardiovascular Medicine and Hypertension, Graduate School of Medical and Dental Sciences, Kagoshima University, Kagoshima, Japan; 2 Department of Cardiology, Fukuoka University School of Medicine, Fukuoka, Japan; 3 Department of Medical Science and Cardiorenal Medicine, Yokohama City University Graduate School of Medicine, Yokohama, Japan; 4 Health Administration Center, University of the Ryukyus, Okinawa, Japan; 5 Division of Public Health, Hygiene and Epidemiology, Tohoku Medical and Pharmaceutical University, Sendai, Japan; 6 Department of Preventive Medicine & Public Health, Fukuoka University School of Medicine, Fukuoka, Japan; 7 Department of Nephrology and Hypertension, Yokohama City University Medical Center, Yokohama, Japan

**Keywords:** Blood pressure, Health personnel, Hypertension, Meta-analysis, Patient care team

## Abstract

Hypertension is a significant risk factor for cardiovascular diseases, with its global prevalence doubling over the past three decades. Despite advancements in antihypertensive therapies, approximately 50% of patients with hypertension fail to achieve their target blood pressure (BP) levels, underscoring the need for innovative care strategies. Patient care teams comprising multidisciplinary healthcare providers have shown promise in improving BP management. This systematic review and meta-analysis were aimed at evaluating the effectiveness of patient care teams involving physicians in hypertension management. To this end, PubMed, Cochrane CENTRAL, and IchuShi-Web were comprehensively searched and 61 randomized controlled trials including 64,857 participants were identified. Compared with usual care, interventions by patient care teams significantly reduced office systolic BP (mean difference: −6.31 mmHg; 95% confidence interval: −7.71 to −4.90) and decreased the risk of uncontrolled BP by 27% (risk ratio: 0.73; 95% confidence interval: 0.68–0.79). Subgroup analyses demonstrated consistent BP reductions across various team leadership roles, such as physicians, nurses, and pharmacists, and across different intervention durations. These findings highlight the effectiveness of team-based BP management in achieving improved BP control, regardless of team composition or the follow-up period. Multidisciplinary care offers a viable approach to addressing the unmet needs of patients with hypertension, potentially improving cardiovascular outcomes. This evidence supports integrating patient care teams into hypertension management, particularly in settings requiring physician oversight. Future research should focus on refining team structures and tailoring interventions to diverse healthcare environments to enhance their impact.

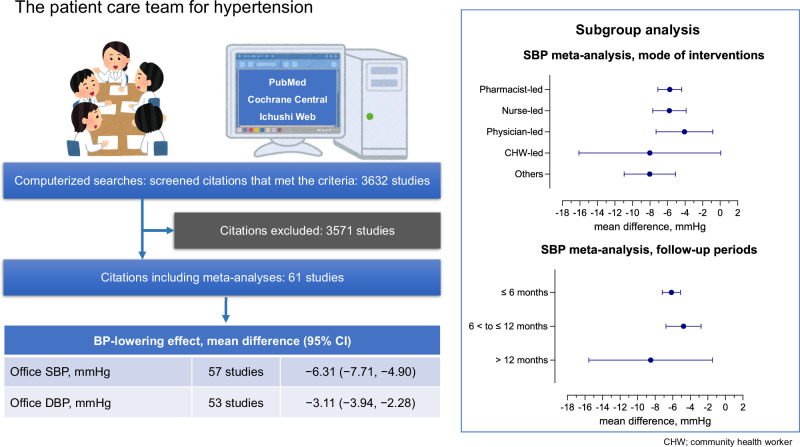

## Introduction

Hypertension is a leading risk factor risk factor for the development of cardiovascular diseases [1], with its global prevalence doubling from 650 million to 1.28 billion over the past three decades [2]. Despite advances in antihypertensive medications, approximately 50% of patients with hypertension fail to achieve their target blood pressure (BP) levels [3]. In Japan, approximately 31 million of the 43 million patients with hypertension did not achieve their BP goals. Of these, 12.5 million had uncontrolled BP despite taking antihypertensive medication [4]. This underscores the pressing need for the development of more efficient therapeutic strategies for treating hypertension.

A patient care team is a collaborative healthcare provider, including physicians, nurses, pharmacists, physician assistants, dieticians, social workers, and community health workers. Although prescribing remains a physician’s duty, task-shifting away from physicians, which is based on collaborative practice agreements with the patient care team, is necessary to meet the enormous demands for the management of hypertension. Furthermore, the patient care team has reportedly been associated with improved BP management compared with the usual physician-only care [5–7]. This model has been recommended by the 2017 American College of Cardiology/American Heart Association blood pressure (BP) guideline and the 2024 European Society of Cardiology Guidelines for the management of elevated BP and hypertension [8, 9]. Interestingly, recent meta-analyses investigating the effectiveness of team-based BP management suggest that the structure of these teams may influence outcomes [7, 10].

Given that patient care teams in Japan operate under physician oversight, we performed a systematic review and meta-analysis to update the evidence of team-based BP management, focusing on patient care teams with physician involvement. We also explored variations in outcomes based on team structure.

## Methods

The authors confirm that all supporting data are provided within the article and its online supplementary materials. This study is a part of the project for creating the 2025 Japanese Society of Hypertension Guidelines for the management of hypertension.

### Search strategy and interventions of interest

The methodology and presentation of this research followed the guidelines of the Preferred Reporting Items for Systematic Reviews and Meta-Analyses (PRISMA) [11]. This systematic review’s comprehensive method and protocol were officially registered with PROSPERO (International Prospective Register of Systematic Reviews) under the identification number CRD42023441440. An extensive literature search was conducted using PubMed, the Cochrane Central Register of Controlled Trials, and IchuShi-Web (a Japanese database) to identify pertinent randomized controlled trials (RCTs) and cluster RCTs up to May 2024. Medical Subject Headings and text keywords related to the patient care team and study type were used (Supplementary Text 1–3).

A patient care team is defined as a team organized under the guidance of a physician and includes at least one or more types of co-medical health workers. The study selection process was limited to research involving human participants and followed predefined inclusion criteria. Data extraction was performed using standardized forms to maintain consistency and thoroughness. The studies included were as follows: (1) Published RCTs or cluster RCTs assessing the impact of patient care teams on BP management in individuals with hypertension; (2) studies for which articles were available in English or Japanese; (3) studies including participants aged 18 years and older; (4) studies in which BP was measured either in office or through noninvasive ambulatory devices; (5) studies providing a clear description of baseline characteristics, changes in office or ambulatory BP, and BP control status, and listing inclusion and exclusion criteria; and (6) studies in which follow-up periods lasted at least 2 months. Studies using interventions by patient care teams across mixed-disease populations were included only if patients with hypertension accounted for more than 50% of the participants. Studies that primarily focused on other conditions, such as diabetes management, were excluded.

Two of the four reviewers (YA, KA, YS, and YS) independently extracted data and screened abstracts and titles of all the retrieved records. After the initial screening, articles, and reviews that might include relevant data were retained. This was followed by a full-text evaluation of all potentially applicable articles. The Medical Information Distribution Service (MINDS) risk of bias assessment tools were used to assess study quality and identify bias risk [12, 13]. Differences in opinion were settled by a discussion with a third reviewer (AS) or through consensus within the systematic review team.

### Outcomes

Outcome measures examined the clinical indicators related to the patient care team’s role in BP management for patients with hypertension, focusing on office or ambulatory systolic and diastolic BP (SBP and DBP) and the status of BP control. The primary outcomes of interest were the pooled mean differences (MDs) in office BP from baseline to each follow-up period (≤6 months, >6–12 months, and >12 months) and the pooled MDs in office or ambulatory BP between the intervention group (BP management in which was performed by the patient care team) and control group (which received usual care). Secondary outcomes included the risk ratios (RRs) of uncontrolled BP across the comparator groups, with definitions of uncontrolled BP taken from each study included in the meta-analysis. The pooled mean differences MDs in office BPs were also compared by age group (<65 years and ≥65 years) and by countries/regions. As shown in Supplementary Table 1, the number of reports from countries other than the U.S. and China/Hong Kong is limited, so they are grouped together under other counties/regions.

### Statistical analysis

The changes in continuous outcomes from baseline to each follow-up period and their standard deviations (SDs) and standard errors (SEs) were recorded along with the participant counts for each group. When only the SE or 95% confidence interval (CI) was reported instead of SD, a standard formula was used for conversion as needed. For any missing parameters essential to the meta-analysis, the Cochrane Handbook for Systematic Reviews of Interventions was used as a reference to make necessary estimations [14]. Before combining the results, risk ratios (RRs) and 95% confidence intervals (CIs) for uncontrolled BP in each trial were calculated. Mean differences (MDs) and RRs were pooled using a random-effects model with inverse variance weighting. The *I*² statistic was used to evaluate the heterogeneity among the studies and to examine the consistency of the results across subgroups classified by intervention [14]. Funnel plots were employed to assess the potential presence of publication bias. The meta-analysis was performed using the Cochrane Collaboration’s Review Manager (RevMan) version 5.4 software. Statistical significance was set at a two-tailed *P*-value of <0.05.

## Results

**Fig. 1:**
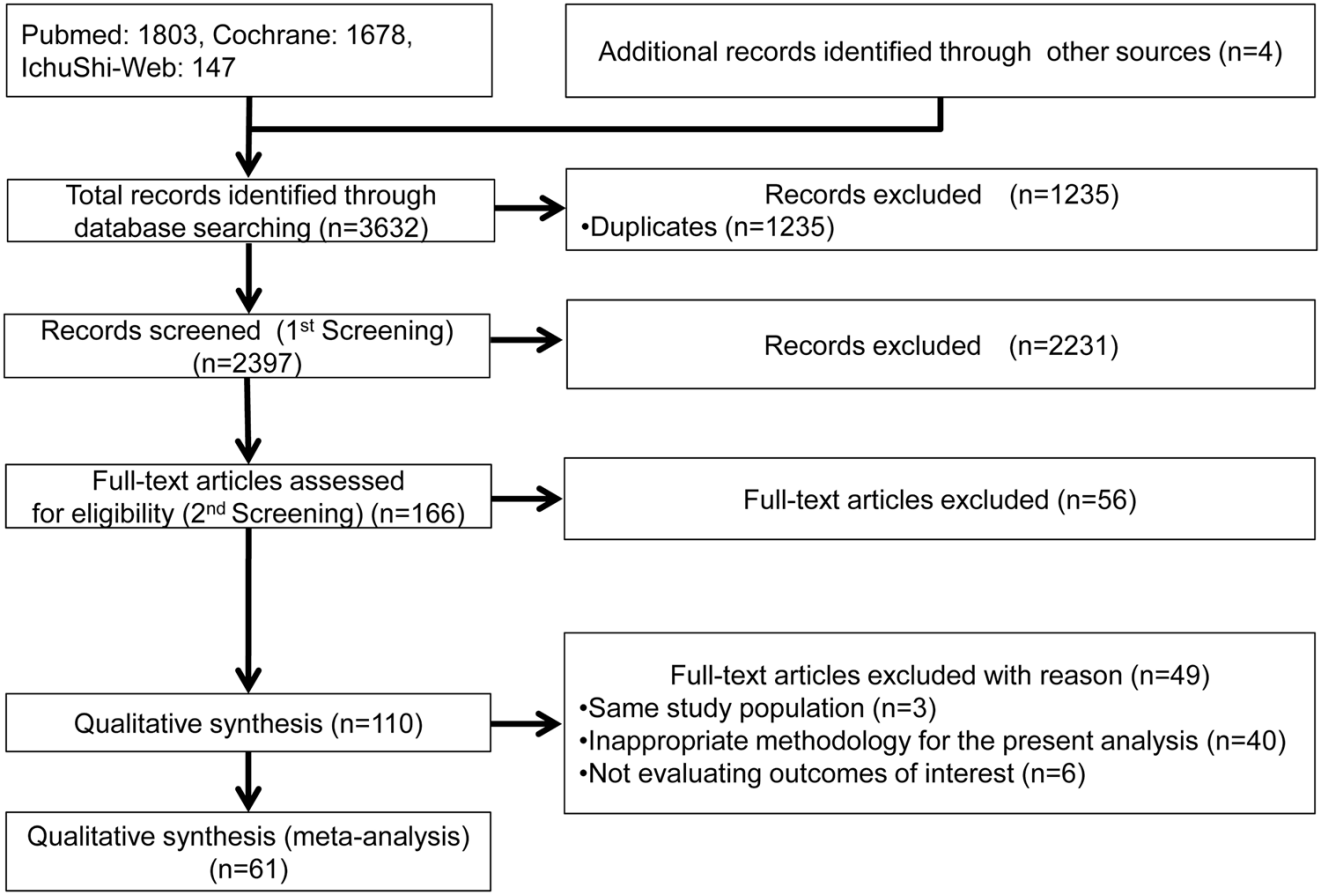
Study selection. The diagram summarizes the search process for relevant studies. 1803 citations were retrieved from PubMed, 1678 from Cochrane CENTRAL, and 147 from IchuShi-Web. Following the screening process, 61 publications were selected for full-text review, all of which were deemed suitable for inclusion in this meta-analysis

Overall, 3632 citations initially met the search criteria. After eliminating 1235 duplicates, 2397 citations were screened. The majority were excluded during the initial abstract review. Subsequently, 166 publications were chosen for a detailed full-text evaluation, with 110 articles excluded owing to specific criteria in the second screening. Finally, 61 publications [15–75] were included in the full-text review and found eligible for this meta-analysis (Fig. 1).

Supplementary Tables 1–2 provide details on the baseline characteristics and office BPs of the patients in the 61 studies included. The meta-analysis encompassed 64,857 participants across these studies (Supplementary Table 1). Four papers did not have data on office BP, and three of the papers were used for analyzing the risk of uncontrolled BP [26, 39, 70] and one for analyzing ambulatory BP monitoring [69]. For a sub-analysis, the studies were divided into the occupations that led the intervention. Interventions were led by pharmacists, nurses, physicians, community health workers, and other patient care workers, including dietitians and health educators, in 22 [15–17, 21, 23, 25, 36, 38, 46, 49, 50, 52, 54, 56, 57, 62, 65–67, 71, 74], 16 [20, 27, 30, 35, 42, 44, 45, 53, 58, 59, 63, 72, 73, 75], 6 [24, 28, 43, 47, 51, 61], 6 [31, 33, 40, 41, 64, 68], and 7 [18, 19, 22, 29, 32, 34, 37] studies, respectively. We defined community health workers are non-physician staff trained in health education programs who provide healthcare support to communities.

In the intervention group, the mean age ranged from 41 to 69 years, and office and ambulatory BP ranges of 127–162/71.9–99.4 mmHg and 133–141/71–88.93 mmHg, respectively (Supplementary Tables 1 and 2). The control group was comparable to the intervention group and had patients with a mean age ranging from 41 to 69.65 years and office and ambulatory BP ranges of 119.18–162/75.13–99 mmHg and 133–140/71–88.16 mmHg, respectively (Supplementary Tables 1–3). The studies varied considerably in their risk of bias, with none showing a low risk for performance bias (Supplementary Table 3).

### Effect of the patient care team on the management of office and ambulatory BP

**Fig. 2:**
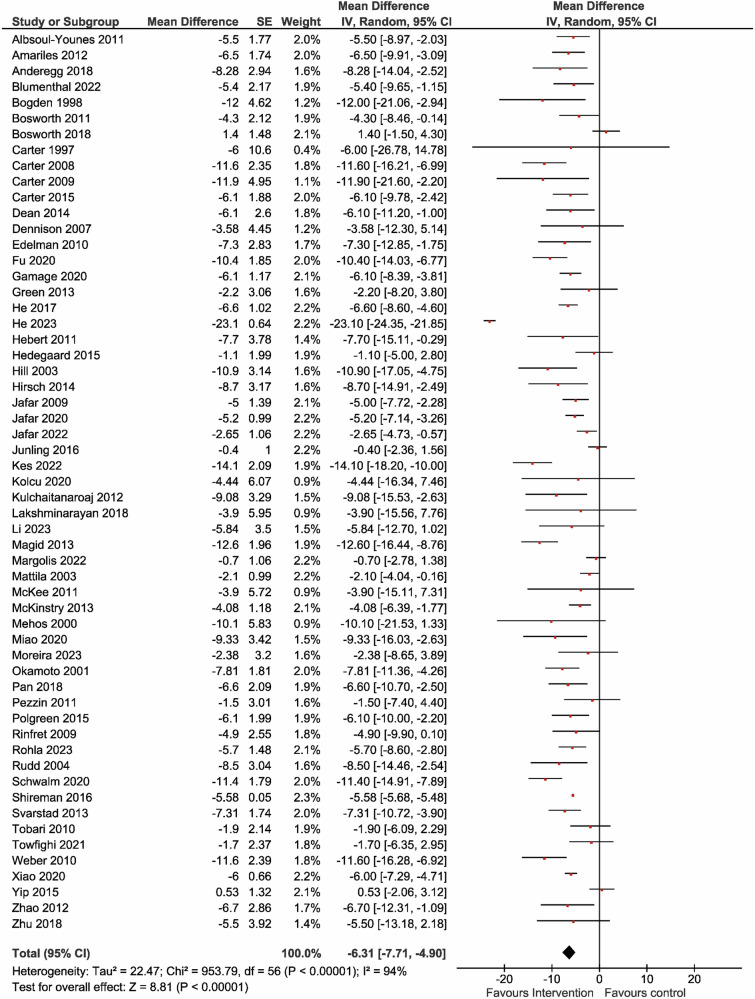
Effect of the patient care team on office systolic blood pressure. The mean difference in systolic blood pressure (SBP) was reported in 57 studies involving 63881 participants. Compared to usual care (control), the intervention provided by the patient care team resulted in a reduction in office SBP (MD = −6.31 [95% CI: −7.71 to −4.90] mmHg), with significant heterogeneity observed (*I*² = 94%). CI confidence interval, IV inverse variance, MD mean difference, SBP systolic blood pressure, SE standard error

**Fig. 3:**
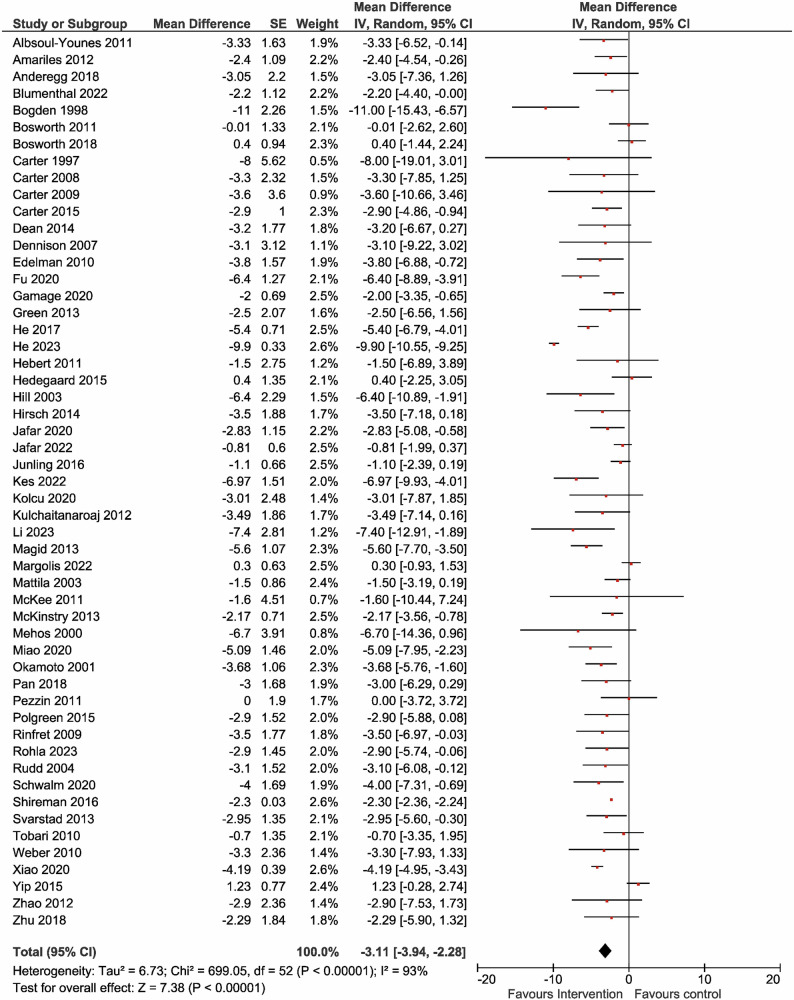
Effect of the patient care team on office diastolic blood pressure. 53 studies involving 62,358 participants reported the mean difference in diastolic blood pressure (DBP). Compared to usual care (control), the patient care team’s intervention reduced office DBP (MD = −3.11 [95% CI: −3.94 to −2.28] mmHg), with significant heterogeneity observed (*I*² = 93%)

Figure 2 shows the MDs in office SBP during the overall intervention period in 63,881 and 62,107 participants in the intervention and control groups, respectively. The between-group differences in the MDs of office SBP varied from −23.1 to 1.4 mmHg. The intervention group showed more significant reductions in office SBP (MD = −6.31 mmHg [95% CI: −7.71 to −4.90]) compared with the control group. The overall effect analysis revealed significant differences in the MDs of office SBP and ambulator SBP between the two groups, with evidence of heterogeneity (Table 1 and Fig. 2). The effects on office DBP and ambulator DBP were also similar to office SBP and ambulator SBP (Table 1 and Fig. 3). Visual inspection of the funnel plot showed no apparent asymmetric distribution of office SBP and DBP (Supplementary Fig. 1A, B). Recognizing that community health workers are rare, the impact of the patient care team on blood pressure was also analyzed without including community health workers. The results indicated that even without community health workers, the overall effect analysis revealed significant differences between the two groups’ MDs of office SBP and office DBP, with evidence of heterogeneity (Supplementary Table 4, Fig. 2D, E).

**Table 1:** Effects of the patient care team for hypertension on the office and ambulatory blood pressure

Outcomes	Trials	Mean difference	Heterogeneity (%)		Test for overall effect	
	*N*	Mean (95% CI)	*I*^2^	*P*-value	*Z*	*P*-value
OSBP (mmHg)	57	−6.31 (−7.71, −4.90)	94	<0.001	8.81	<0.001
ODBP (mmHg)	53	−3.11 (−3.94, −2.28)	93	<0.001	7.38	<0.001
ASBP (mmHg)	8	−8.11 (−10.96, −5.27)	72	0.001	5.59	<0.001
ADBP (mmHg)	6	−3.53 (−5.08, −1.97)	54	0.05	4.45	<0.001

*ADBP* ambulatory diastolic blood pressure, *ASBP* ambulatory systolic blood pressure, *CI* confidence interval, *N* number, *ODBP* office diastolic blood pressure, *OSBP* office systolic blood pressure

Comparable effects of the interventions on office SBP were observed during each follow-up period (Table 2). The observed beneficial effect of the patient care team for BP management on office BP was evaluated according to the baseline characteristics of the studies (Table 2). Table 2 shows the impact of the intervention on SBP in the subgroups defined by the leading professionals. The reduction in SBP did not significantly differ according to the professionals leading the interventions. In the analysis by follow-up period, BP reduction was also observed for periods of less than 6 months, 6–12 months, and more than 12 months (Table 2). For analyses, values from the most extended follow-up periods were used when multiple office and ambulatory BP measurements were available. The intervention group showed significantly more significant reductions in office SBP compared with the control group (Table 2). We analyzed the impact of the patient care team on office SBP across two age groups: those under 65 years and those aged 65 years and older. Our findings indicated that there was no significant difference in SBP reduction between these age groups (Table 2). Additionally, the reduction in office SBP did not vary significantly between different countries or regions (Table 2).

**Table 2:** Effects of the patient care team for hypertension on office systolic blood pressure in subgroups

Subgroups	Trials	Mean difference (mmHg)	Heterogeneity (%)		Test for overall effect		*P*-value for heterogeneity
	*N*	Mean (95% CI)	*I*^2^	P-value	*Z*	*P*-value	*P*-value
Intervention							0.46
Pharmacist-led	22	−5.74 (−7.10, −4.38)	74	<0.001	8.27	<0.001	
Nurse-led	16	−5.79 (−7.69, −3.88)	74	<0.001	5.96	<0.001	
Physician-led	6	−4.07 (−7.31, −0.83)	76	<0.001	2.46	<0.001	
CHW-led	6	−8.02 (−16.10, 0.07)	99	<0.001	1.94	0.05	
Others	7	−8.03 (−10.95, −5.10)	42	0.11	5.37	<0.001	
Follow-up periods							0.37
≤6 months	33	−6.15 (−7.20, −5.11)	59	<0.001	11.55	<0.001	
>6 to ≤12 months	16	−4.77 (−6.77, −2.77)	84	<0.001	4.68	<0.001	
>12 months	8	−8.51 (−15.55, −1.48)	99	<0.001	2.37	0.02	
Mean age							0.89
<65 years	47	−6.27 (−7.85, −4.69)	95	<0.001	7.79	<0.001	
≥65 years	10	−6.53 (−9.94, −3.11)	81	<0.001	3.75	<0.001	
Countries/regions							0.59
United States	30	−6.24 (−7.55, −4.93)	68	<0.001	9.32	<0.001	
China/Hong Kong	10	−7.36 (−14.08, −0.65)	99	<0.001	2.15	0.03	
Other countries/regions	17	−5.34 (−6.72, −3.95)	72	<0.001	7.56	<0.001	

*CHW* community health worker, *CI* confidence interval, *HT* hypertension, *N* number

### Effect of the patient care team for BP management on the risk of uncontrolled BP

**Fig. 4:**
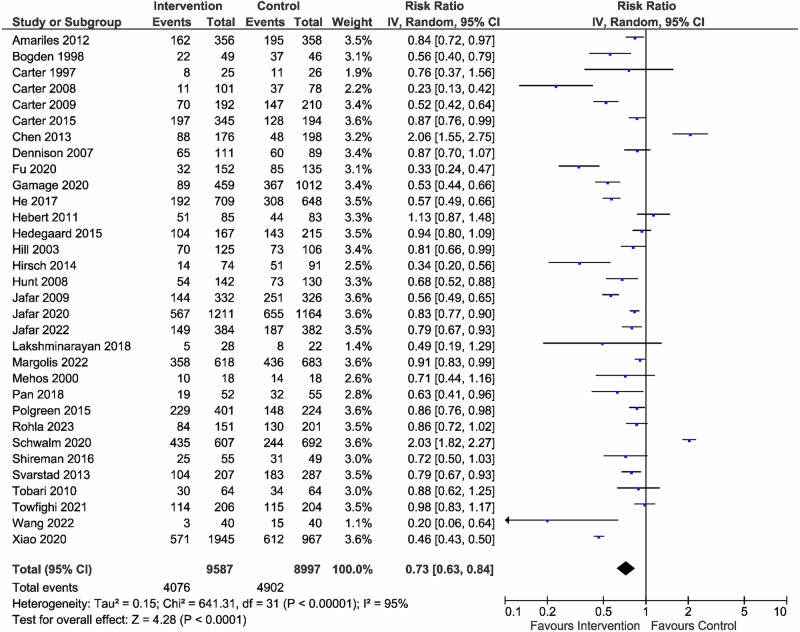
Effect of the patient care team on risk for uncontrolled BP. The risk ratio for uncontrolled BP was reported in 32 studies involving 18,584 participants. Compared to usual care (control), the intervention provided by the patient care team resulted in a risk reduction of uncontrolled BP (Risk Ratio = 0.73 [95% CI: 0.63–0.84]), with significant heterogeneity observed (*I*² = 95%)

Figure 4 shows the RRs of uncontrolled BP in 10,748 and 10,147 participants in the intervention and control groups, respectively. Interventions reduced the risk of uncontrolled BP by 27% compared with usual care (*RR* = 0.73, 95% CI: 0.68–0.79), with evidence of heterogeneity. Our analysis suggests the potential presence of publication bias in this meta-analysis, as evidenced by the reduced number of studies (Supplementary Fig. 1C). This bias may have led to overestimating the BP control results. Interventions also reduced the risk of uncontrolled BP by 24% compared with usual care, even without community health workers (Supplementary Fig. 5).

## Discussion

This meta-analysis of evidence synthesized from 61 studies highlights the significant benefits of patient care teams involving physicians in improving BP control among patients with hypertension. Patient care teams involving physicians achieved a significant reduction in office SBP and DBP. Additionally, interventions by these teams led to a significant decrease in SBP, irrespective of the professionals leading the teams or the duration of the intervention, with evidence of heterogeneity. Furthermore, the patient care teams significantly reduced office DBP and lowered the rate of uncontrolled BP.

These results indicate a definite role for patient care teams involving physicians in BP reduction among patients with hypertension. Patient care teams for BP management are multidisciplinary teams, including physicians, nurses, pharmacists, physician assistants, dieticians, social workers, and community health workers for prevention and control of hypertension [76]. Patients’ medical conditions, socioeconomic status, medication adherence, presence of family support, nutritional status, and decision-making are shared with these teams. Each professional in the team has solid points for hypertension control. The physicians on these teams mainly focus on pathological conditions, medication-related decisions regarding the effect of drugs and medication adherence, and patients’ socioeconomic status. Creating an environment for success, sharing quality data, and promoting care team collaboration are also central roles of these physicians [77]. Nurses are mainly involved in total management, patient counseling, support disease awareness, care coordination, population health management, and performance evaluation [78]. Pharmacists are mainly focused on total medicine management, medication adherence, disease state education, and patient counseling [79]. In addition to the contribution of each specialty toward disease management, frequent contact by each professional on the team helps understand disease awareness; notice the importance of daily management, including meals, exercise, and medication; and leads to hypertension control [76].

In this study, community health worker-led (CHW-led) interventions resulted in a −8.03 mmHg reduction in the office SBP. However, the heterogeneity of each professional-led intervention was not significant, and the reduction achieved by CHW-led interventions was large compared with those achieved by interventions led by other professionals. A similar finding was reported by Mills et al.; they stated that CHW-led interventions led to a −7.1 mmHg reduction in SBP and the reduction was large compared with those achieved by physician- or nurse-led interventions [80]. Community health workers provide services pertaining to lifestyle modification, medication adherence, and training for home BP monitoring to patients and families and serve as mediators between patients and physicians. They also arrange physician appointments, motivate patients and families, and provide social support [33]. Patient care teams with community health workers would be more useful for BP management because physicians have regulated working hours owing to the promotion of working style reforms in Japan.

The duration of team intervention is an essential point for hypertension control. In patients with hypertension, prolonged BP control is necessary [81]. In this study, we divided the duration into three periods: less than 6 months, 6–12 months, and 12 months and more. The difference in duration did not affect the reduction of office SBP among patients with hypertension, with evidence of heterogeneity.

Patient care teams involving physicians achieved a reduction in office SBP and DBP, 24-h ambulatory SBP and DBP, and the percentage of controlled hypertension. Thus, the patient care team with the physician demonstrated better BP control in a variety of outcomes. The strengths of the study are as follows: use of a standardized methodology as documented in PRISMA [11] and MINDS [13] under the Cochrane guidelines for conducting systematic reviews and meta-analyses [14], the inclusion of large sample size with varying demographic and cultural characteristics, and the sensitivity assessment by subgroup analyses. However, it also has several limitations. First, this meta-analysis did not include unpublished data owing to limited access. Second, there was heterogeneity. The variations in sample size, leading professionals, study duration, and the presence of digital health interventions are likely limitations. It will be necessary to study which team is effective in controlling hypertension. To address this issue, a long-term follow-up study extending for more than 2 years will be required because most studies conducted thus were shorter than 1 year. Patient education is also effective in controlling hypertension. The long-term effect of short-term interventions for maintaining BP must be investigated. Furthermore, the effects of interventions by patient care teams on cardiovascular events, body mass index, behavior change, medication adherence, quality of life, and cost-effectiveness should be investigated in the future. In addition, no analysis of medication adherence was possible because no data was collected. Further analysis regarding social security and medical insurance systems should be considered in the future.

In conclusion, patient care teams involving physicians significantly reduced BP and the percentage of uncontrolled hypertension in patients with hypertension. These findings highlight the effectiveness of team-based BP management in achieving improved BP control, regardless of team composition or follow-up period. Multidisciplinary care offers a viable approach to addressing the unmet needs of patients with hypertension, potentially improving cardiovascular outcomes. This evidence supports integrating patient care teams into hypertension management, particularly in settings requiring physician oversight. Future research should focus on refining team structures and tailoring interventions to diverse healthcare environments to enhance their impact.

## Supplementary information

Supplementary Information
