# Supplementary material for: Impact of patient care teams on blood pressure control in patients with hypertension: a systematic review and meta-analysis
Source: Hypertens Res. 2025 Feb 17;48(6):1827–38. doi: 10.1038/s41440-025-02152-9 (PMC12137128; doi:10.1038/s41440-025-02152-9)
Supplement: Supplementary file 1 — Supplementary Information [file 41440_2025_2152_MOESM1_ESM.docx]

**Supplementary Content**

Supplemental to:

**Impact of Patient Care Teams on Blood Pressure Control in Patients with Hypertension: A Systematic Review and Meta-Analysis**

1. Table S1: Baseline characteristics of the studies included in the meta-analysis
2. Table S2: Baseline office blood pressure of the studies included in the meta-analysis
3. Table S3: Summary of risk of bias assessments among included studies
4. Table S4: Effects of the patient care team other than community health worker-led (CHW-led) care for hypertension on the office blood pressure
5. Table S5: Effects of the patient care team other than CHW-led care for hypertension on office systolic blood pressure in subgroups
6. Figure S1: Funnel plots of effects of patient care team for hypertension on office blood pressure and risk for uncontrolled blood pressure
7. Figure S2: Funnel plots of effects of the patient care team other than CHW-led care for hypertension on the office blood pressure, and risk for uncontrolled blood pressure
8. Figure S3: Effects of the patient care team other than CHW-led care for hypertension on the office systolic blood pressure
9. Figure S4: Effects of the patient care team other than CHW-led care for hypertension on the office diastolic blood pressure
10. Figure S5: Effects of the patient care team other than CHW-led care for hypertensionon the risk for uncontrolled blood pressure
11. Text S1: Search strategy in PubMed
12. Text S2: Search strategy in Cochrane Central
13. Text S3: Search strategy in IchuShi Web

# **Supplementary Table 1: Baseline characteristics of the studies included in the meta-analysis**

| Author and publication year | Country/region | Mean age, years | | Sample size, N | | Follow-up periods | Mode of intervention |
| --- | --- | --- | --- | --- | --- | --- | --- |
|  |  | Intervention | Control | Intervention | Control |  |  |
| Albsoul-Younes (2011) ^15)^ | Saudi Arabia | 56.3±9.6 | 57.5±11.9 | 130 | 123 | 6 months | Pharmacist-led |
| Amariles (2012) ^16)^ | Spain | 63±8.3 | 62.6±8 | 356 | 358 | 8 months | Pharmacist-led |
| Anderegg (2018) ^17)^ | USA | 61.7±11.6 | 63.1±12.2 | 227 | 108 | 9 months | Pharmacist-led |
| Blumenthal (2022) ^18)^ | USA | 62±9 | 63±9 | 90 | 50 | 4 months | Others |
| Bogden (1998) ^19)^ | USA | 56±13 | 54±13 | 49 | 46 | 6 months | Others |
| Bosworth (2011) ^20)^ | USA | 63±11 | 64±10 | 147 | 147 | 1 year | Nurse-led |
| Bosworth (2018) ^21)^ | USA | 60.9±8.4 | 61.5±8.9 | 215 | 213 | 12 months | Pharmacist-led |
| Carter (1997) ^22)^ | USA | 67.3 | 68.5 | 25 | 26 | 6 months | Pharmacist-led |
| Carter (2008) ^23)^ | USA | 59.6±13.7 | 61.9±11.3 | 101 | 78 | 9 months | Pharmacist-led |
| Carter (2009) ^24)^ | USA | 57.3±14.3 | 59.2±13.8 | 192 | 210 | 6 months | Pharmacist-led |
| Carter (2015) ^25)^ | USA | 61.8±12.4 | 61.8±13.7 | 401 | 224 | 9 months | Physician-led |
| Chen (2013) ^26)^ | USA | 57.2±14.5 | 59.4±14 | 176 | 198 | 6 months | Physician-led |
| Dean (2014) ^27)^ | United Kingdom | 62±14 | 62±13 | 167 | 186 | 6 months | Others |
| Dennison (2007) ^28)^ | USA | 41±6 (overall) | | 157 | 152 | 5 years | Nurse-led |
| Edelman (2010) ^29)^ | USA | 63±10.2 | 60.8±10 | 133 | 106 | 1 year | Physician-led |
| Fu (2020) ^30)^ | Hong Kong | 67.8± | 66.1±9.7 | 137 | 152 | 18 months | CHW-led |
| Gamage (2020) ^31)^ | India | 56.6±14.3 | 56.9±13.7 | 637 | 1097 | 2 months | Others |
| Green (2013) ^32)^ | USA | 55.9±7.2 | 57.8±6.7 | 27 | 15 | 6 months | CHW-led |
| He (2017) ^33)^ | Argentina | 56.1±13.6 | 55.5±13 | 743 | 689 | ≥16 months | Nurse-led |
| He (2023) ^34)^ | China | 62.8±9.2 | 63.2±9.2 | 17407 | 16588 | 3 months | Pharmacist-led |
| Hebert (2012) ^35)^ | USA | 60.5±11.1 | 61.2±12 | 120 | 118 | 9 months | Pharmacist-led |
| Hedegaard (2015) ^36)^ | Denmark | 62 (54-68) | 60 (52-68) | 231 | 285 | 1 year | Pharmacist-led |
| Hill (2003) ^37)^ | USA | 41±6 (overall) | | 125 | 106 | 3 years | CHW-led |
| Hirsch (2014) ^38)^ | USA | 65.4±13 | 69.65±11.4 | 75 | 91 | 6 months | Nurse-led |
| Hunt (2008) ^39)^ | USA | 68±12 | 68±13 | 230 | 233 | 1 year | CHW-led |
| Jafar (2009) ^40)^ | Pakistan | 54±11.5 | 53.3±11.5 | 332 | 336 | 2 years | CHW-led |
| Jafar (2020) ^41)^ | Bangladesh, Pakistan, Sri Lanka | 58.5±11.2 | 59±11.8 | 1330 | 1315 | 24 months | Physician-led |
| Jafar (2022) ^42)^ | Singapore | 63±9.7 | 65.9±9.7 | 447 | 469 | 2 years | Nurse-led |
| Junling (2015) ^43)^ | China | 66±9.3 | 67.1±10.3 | 600 | 604 | 6 months | Nurse-led |
| Kes (2022) ^44)^ | Turkey | 54.9±6.6 | 52.2±6.2 | 46 | 46 | 12 weeks | Pharmacist-led |
| Kolcu (2020) ^45)^ | Turkey | 75.63±7.25 (overall) | | 37 | 37 | 6 months | Physician-led |
| Kulchaitanaroaj (2012) ^46)^ | USA | 59.1±13.7 | 61.3±12.9 | 252 | 244 | 6 months | Pharmacist-led |
| Lakshminarayan (2018) ^47)^ | USA | 63.1±9.7 | 68.3±10 | 34 | 22 | 3 months | Pharmacist-led |
| Li (2023) ^48)^ | China | 64.06±9.43 | 63.42±9.06 | 51 | 50 | 12 months | Pharmacist-led |
| Magid (2013) ^49)^ | USA | 60±11.3 | 59.1±10.9 | 175 | 173 | 6 months | Pharmacist-led |
| Margolis (2022) ^50)^ | USA | 62.4±14.2 | 58.3±14.2 | 1423 | 1648 | 1 year | Others |
| Mattila (2003) ^51)^ | Finland | 49.9±5.85 | 49.8±6.33 | 356 | 347 | 12 months | Physician-led |
| McKee (2011)^52)^ | USA | 61.2±11.2 | 58.6±7.9 | 31 | 24 | 6 months | Nurse-led |
| McKinstry (2013) ^53)^ | Scotland | 60.5±11.8 | 60.8±10.7 | 200 | 201 | 6 months | Nurse-led |
| Mehos (2000) ^54)^ | USA | 60±14.8 | 57.6±13.5 | 18 | 18 | 6 months | Nurse-led |
| Miao (2020) ^55)^ | China | 68.9±8.9 | 66.8±9.2 | 78 | 78 | 12 weeks | Pharmacist-led |
| Moreira (2023) ^56)^ | Brazil | 66.88 | 66.85 | 161 | 161 | ≥6 weeks | Nurse-led |
| Okamoto (2001) ^57)^ | USA | 61.95±11.4 | 61.71±11.3 | 164 | 166 | 6 months | Others |
| Pan (2018) ^58)^ | China | 56.55±9.8 | 57.8±10.87 | 52 | 55 | 6 months | Nurse-led |
| Pezzin (2011) ^59)^ | USA | 64.2±11.1 | 64.3±10.5 | 221 | 217 | 3 months | Nurse-led |
| Polgreen (2015) ^60)^ | USA | 61±1.01 (overall) | | 401 | 224 | 9 months | Pharmacist-led |
| Rinfret (2009) ^61)^ | USA | 55±11 | 57±13 | 111 | 112 | ≥4 months | Physician-led |
| Rohla (2023) ^62)^ | Austria | 68±8 | 66±14 | 207 | 290 | 5 months | Pharmacist-led |
| Rudd (2004) ^63)^ | USA | 59±10 | 60±9 | 74 | 76 | 6 months | Nurse-led |
| Schwalm (2020) ^64)^ | Colombia, Malaysia | 65.1±9.1 | 65.8±9.7 | 644 | 727 | 1 year | Others |
| Shireman (2016) ^65)^ | USA | 54.1±10.5 | 52.8±11.9 | 287 | 207 | 6 months | Pharmacist-led |
| Svarstad (2013) ^66)^ | USA | 53.2±11 | 52.8±11.9 | 276 | 300 | 6 months | Pharmacist-led |
| Tobari (2010) ^67)^ | Japan | 61.7±6.9 | 61.6±8.5 | 66 | 66 | 6 months | Pharmacist-led |
| Towfighi (2021) ^68)^ | USA | 57.2±9 | 57±8.7 | 241 | 246 | 12 months | CHW-led |
| Wang (2011) ^69)^ | China | 47.23±7.69 | 48.3±9.06 | 29 | 30 | 12 months | Pharmacist-led |
| Wang (2022) ^70)^ | China | 42 | 43 | 40 | 40 | 3 months | Pharmacist-led |
| Weber (2010) ^71)^ | USA | 59.6±13.7 | 61.9±11.3 | 101 | 78 | 9 months | Pharmacist-led |
| Xiao (2020) ^72)^ | China | 57.72±7.5 | 58.05±8.86 | 1945 | 967 | 1 year | Nurse-led |
| Yip (2015) ^73)^ | Hong Kong | 64±9.1 | 62.9±8.3 | 194 | 199 | 1 year | Nurse-led |
| Zhao (2012) ^74)^ | China | 62.4±19.1 | 65.6±18.8 | 139 | 139 | 6 months | Pharmacist-led |
| Zhu (2018) ^75)^ | China | 69±9.5 | 69±10.2 | 67 | 67 | 16 weeks | Nurse-led |

Data are expressed as the mean ± SD, mean, median (IQR), number and category.

Abbreviations: CHW: community health worker; ICQ: interquartile range; N: number; SD: standard deviation.

# **Supplementary Table 2: Baseline office blood pressure of the studies included in the meta-analysis**

| Author and publication year | Office SBP, mmHg | | Office DBP, mmHg | |
| --- | --- | --- | --- | --- |
|  | Intervention | Control | Intervention | Control |
| Albsoul-Younes (2011) ^15)^ | 137.5±15.4 | 134.8±14.6 | 85.2±8.7 | 83.7±8.1 |
| Amariles (2012) ^16)^ | 145±17.0 | 143±17.6 | 83.3±10.4 | 82±11.3 |
| Anderegg (2018) ^17)^ | 147±15.71 | 147.36±16.26 | 81.82±11.65 | 80.7±12.09 |
| Blumenthal (2022) ^18)^ | 139±10 | 140±10 | 79±9 | 80±8 |
| Bogden (1998) ^19)^ | 155±24 | 156±18 | 96±8 | 95±10 |
| Bosworth (2011) ^20)^ | 127±21 | 128±17 | 77±13 | 78±14 |
| Bosworth (2018) ^21)^ | 130.6±18.5 | 129.7±18.8 | 75.8±11.5 | 75.8±12.4 |
| Carter (1997) ^22)^ | 146±24 | 147±24 | 83±14 | 82±11 |
| Carter (2008) ^23)^ | 153.1±10 | 150.3±9 | 84.9±12 | 85.4±11 |
| Carter (2009) ^24)^ | 153.6±12.8 | 150.6±14.1 | 87.4±11.9 | 83.6±12.3 |
| Carter (2015) ^25)^ | 147.6±13.7 | 149.6±15.3 | 83.5±12.4 | 84.3±12.6 |
| Chen (2013) ^26)^ | 135±15.4 (ABPM) | 137.0±16.3 (ABPM) | N/A | N/A |
| Dean (2014) ^27)^ | 155±18 | 153±14 | 86±11 | 86±12 |
| Dennison (2007) ^28)^ | 147±19.4 | 148±20.9 | 99±14.5 | 99±14.9 |
| Edelman (2010) ^29)^ | 153.7±14.8 | 151.9±13.4 | 84.7±12.1 | 84.2±13.8 |
| Fu (2020) ^30)^ | 152±9.87 | 152±10.4 | 78.5±10.7 | 80.76±11.9 |
| Gamage (2020) ^31)^ | 140.5±22.7 | 137.8±22.2 | 80.4±13.7 | 80.6±13.9 |
| Green (2013) ^32)^ | 150.1±11.6 | 150.6±11.9 | 92.6±8.8 | 90.7±9.5 |
| He (2017) ^33)^ | 151.7±16.8 | 149.8±15.5 | 92.2±12.2 | 90.1±12.9 |
| He (2023) ^34)^ | 157±18 | 155.4±17.3 | 87.9±10.7 | 87.2±10.6 |
| Hebert (2012) ^35)^ | 152±13.4 | 152±15.3 | 75.2±14 | 77.1±13.7 |
| Hedegaard (2015) ^36)^ | 136.8±15.6 | 136.6±16.3 | 78.1±9.9 | 78.3±12.1 |
| Hill (2003) ^37)^ | 146.8±19.4 | 147.5±20.9 | 99.4±14.5 | 98.5±14.9 |
| Hirsch (2014) ^38)^ | 134.8±17.4 | 134.4±16.5 | 75.1±12.5 | 75.7±13.4 |
| Hunt (2008) ^39)^ | 173±15 | 174±15 | 90±14 | 92±14 |
| Jafar (2009) ^40)^ | 148.3±24.7 | 153.3±24.6 | 91.1±13 | 95.5±12.5 |
| Jafar (2020) ^41)^ | 146.7±22.4 | 144.7±21 | 89.1±14.7 | 87.8±13.8 |
| Jafar (2022) ^42)^ | 148.4±11.8 | 150.1±14 | 89.4±9.2 | 87.3±10.2 |
| Junling (2015) ^43)^ | 139.2±13.4 | 139.2±12.5 | 85.3±8.5 | 84.9±7.9 |
| Kes (2022) ^44)^ | 156.26±5.14 | 155.32±5.67 | 95.08±4.22 | 94.16±4.66 |
| Kolcu (2020) ^45)^ | 129.18±14.6 | 119.18±15.16 | 79.72±9.57 | 75.13±10.17 |
| Kulchaitanaroaj (2012) ^46)^ | 153.5±11.8 | 150.8±12.7 | 86.4±11.7 | 83.1±11.9 |
| Lakshminarayan (2018) ^47)^ | 140±15.7 | 139.8±16.3 | N/A | N/A |
| Li (2023) ^48)^ | 144.98±14.78 | 143.78±13.95 | 83.55±11.21 | 81.66±12.39 |
| Magid (2013) ^49)^ | 148.8±16.2 | 145.5±14.5 | 89.6±10.2 | 88±9.9 |
| Margolis (2022) ^50)^ | 158.8±15.2 | 157.4±15.4 | 90±13.8 | 93.1±13.8 |
| Mattila (2003) ^51)^ | 139.5±14.7 | 137.4±14.2 | 90.5±9.02 | 89.1±8.76 |
| McKee (2011)^52)^ | 138.6±14.1 | 143.1±14.6 | 71.9±10.1 | 76.6±13.9 |
| McKinstry (2013) ^53)^ | 146±10.5 | 146.5±10.7 | 87.4±10.1 | 83.4±9.1 |
| Mehos (2000) ^54)^ | 157.9±16.4 | 153.9±14.6 | 91.1±10.8 | 89.6±9.8 |
| Miao (2020) ^55)^ | N/A | N/A | N/A | N/A |
| Moreira (2023) ^56)^ | 142.07±22 | 144.27±23.56 | 93.16 | 90.51 |
| Okamoto (2001) ^57)^ | 144.23±18.4 | 142.91±18 | 82.79±11.2 | 82.13±11.4 |
| Pan (2018) ^58)^ | 148.3±7.1 | 147.9±8.7 | 88±7.5 | 87±8.4 |
| Pezzin (2011) ^59)^ | 154.3±20.1 | 156.1±20.2 | 86.8±12.2 | 88.1±16 |
| Polgreen (2015) ^60)^ | 148.9±14.8 | 149.7±15.3 | 85.1±12.1 | 84.3±12.6 |
| Rinfret (2009) ^61)^ | 162±16 | 162±17 | 91±12 | 90±12 |
| Rohla (2023) ^62)^ | 150±13 | 148±13 | 85±8 | 85±9 |
| Rudd (2004) ^63)^ | N/A | N/A | N/A | N/A |
| Schwalm (2020) ^64)^ | 152.1±15.4 | 151.8±15.6 | 84.7±12 | 85.3±11.9 |
| Shireman (2016) ^65)^ | 151.2±15.2 | 153.1±16.6 | 92±10.1 | 92.9±10 |
| Svarstad (2013) ^66)^ | 151.2±15.2 | 153.1±16.6 | 92±10.1 | 92.9±10 |
| Tobari (2010) ^67)^ | 138±12.43 | 139±12.43 | 81±6.26 | 83±8.29 |
| Towfighi (2021) ^68)^ | 143.2±17.1 | 145.7±18.6 | N/A | N/A |
| Wang (2011) ^69)^ | 145.60±7.34 | 143.45±7.33 | 95.50±8.67 | 94.30±6.59 |
| Wang (2022) ^70)^ | N/A | N/A | N/A | N/A |
| Weber (2010) ^71)^ | 153.1±10 | 150.3±9 | 84.9±12 | 85.4±11 |
| Xiao (2020) ^72)^ | 150.4±11.39 | 151.1±17.39 | 94.45±7.24 | 94.64±8.48 |
| Yip (2015) ^73)^ | 123.8±9.7 | 123.4±10.8 | 73.1±9.4 | 72.3±9 |
| Zhao (2012) ^74)^ | 142.5±16.6 | 143.9±17.2 | 85.2±10.2 | 86.4±11.7 |
| Zhu (2018) ^75)^ | 153.9±16.74 | 149.65±14.59 | 82.63±11.47 | 83.53±11.45 |

Data are expressed as the mean ± SD.

Abbreviations: ABPM; ambulatory blood pressure monitoring; DBP: diastolic blood pressure; N/A: not applicable; SBP: systolic blood pressure; SD: standard deviation.

# **Supplementary Table 3: Summary of risk of bias assessments among included studies**

| Individual research | | Risk of bias | | | | | |
| --- | --- | --- | --- | --- | --- | --- | --- |
| Study | Design | R | D | Mi | Me | S | O |
| Albsoul-Younes (2011) ^15)^ | RCT | Low | 0 | -1 | 0 | 0 | 0 |
| Amariles (2012) ^16)^ | RCT | 0 | 0 | -1 | -1 | 0 | 0 |
| Anderegg (2018) ^17)^ | RCT | 0 | -1 | -1 | -1 | 0 | -1 |
| Blumenthal (2022) ^18)^ | RCT | 0 | -1 | 0 | 0 | 0 | 0 |
| Bogden (1998) ^19)^ | RCT | 0 | -1 | 0 | 0 | 0 | 0 |
| Bosworth (2011) ^20)^ | RCT | 0 | -1 | -1 | -1 | 0 | -1 |
| Bosworth (2018) ^21)^ | RCT | 0 | -1 | -1 | -1 | 0 | -1 |
| Carter (1997) ^22)^ | RCT | 0 | -1 | 0 | 0 | 0 | 0 |
| Carter (2008) ^23)^ | CRCT | -1 | -1 | 0 | 0 | 0 | 0 |
| Carter (2009) ^24)^ | CRCT | 0 | -1 | -1 | -1 | 0 | -1 |
| Carter (2015) ^25)^ | RCT | 0 | -1 | 0 | -1 | 0 | 0 |
| Chen (2013) ^26)^ | CRCT | 0 | -1 | 0 | -1 | 0 | 0 |
| Dean (2014) ^27)^ | RCT | 0 | 0 | -1 | -1 | 0 | 0 |
| Dennison (2007) ^28)^ | RCT | -1 | -1 | -1 | 0 | 0 | 0 |
| Edelman (2010) ^29)^ | RCT | 0 | -1 | 0 | 0 | 0 | 0 |
| Fu (2020) ^30)^ | RCT | 0 | -1 | 0 | -1 | 0 | 0 |
| Gamage (2020) ^31)^ | RCT | 0 | -1 | 0 | -1 | 0 | 0 |
| Green (2013) ^32)^ | RCT | 0 | 0 | -1 | 0 | 0 | 0 |
| He (2017) ^33)^ | CRCT | 0 | -1 | -1 | -1 | 0 | -1 |
| He (2023) ^34)^ | CRCT | 0 | -1 | 0 | -1 | 0 | 0 |
| Hebert (2011) ^35)^ | RCT | 0 | 0 | -1 | 0 | 0 | 0 |
| Hedegaard (2015) ^36)^ | RCT | 0 | -1 | 0 | 0 | 0 | 0 |
| Hill (2003) ^37)^ | RCT | 0 | -1 | 0 | -1 | -1 | -1 |
| Hirsch (2014) ^38)^ | RCT | 0 | -1 | -1 | -1 | 0 | -1 |
| Hunt (2008) ^39)^ | RCT | 0 | -1 | -1 | 0 | 0 | -1 |
| Jafar (2009) ^40)^ | CRCT | 0 | 0 | 0 | 0 | 0 | 0 |
| Jafar (2020) ^41)^ | RCT | 0 | 0 | 0 | -1 | 0 | 0 |
| Jafar (2022) ^42)^ | CRCT | 0 | -1 | 0 | -1 | 0 | 0 |
| Junling (2015) ^43)^ | RCT | -1 | -1 | 0 | -1 | 0 | -1 |
| Kes (2022) ^44)^ | RCT | -1 | 0 | -1 | 0 | 0 | 0 |
| Kolcu (2020) ^45)^ | CRCT | 0 | -1 | 0 | -1 | 0 | 0 |
| Kulchaitanaroaj (2012) ^46)^ | CRCT | 0 | -1 | 0 | -1 | 0 | 0 |
| Lakshminarayan (2018) ^47)^ | RCT | 0 | -1 | 0 | -1 | 0 | 0 |
| Li (2023) ^48)^ | RCT | 0 | -1 | -1 | -1 | 0 | -1 |
| Magid (2013) ^49)^ | RCT | 0 | -1 | -1 | 0 | 0 | 0 |
| Margolis (2022) ^50)^ | CRCT | 0 | -1 | -1 | -1 | 0 | -1 |
| Mattila (2003) ^51)^ | RCT | 0 | -1 | -1 | 0 | 0 | 0 |
| McKee (2011) ^52)^ | RCT | -1 | -1 | -1 | 0 | 0 | 0 |
| McKinstry (2013) ^53)^ | RCT | 0 | -1 | 0 | -1 | 0 | 0 |
| Mehos (2000) ^54)^ | RCT | 0 | -1 | 0 | -1 | 0 | 0 |
| Miao (2020) ^55)^ | RCT | 0 | -1 | 0 | 0 | 0 | 0 |
| Moreira (2023) ^56)^ | RCT | 0 | -1 | 0 | -1 | 0 | 0 |
| Okamoto (2001) ^57)^ | RCT | 0 | -1 | 0 | -1 | 0 | 0 |
| Pan (2018) ^58)^ | RCT | 0 | -1 | 0 | -1 | 0 | 0 |
| Pezzin (2011) ^59)^ | RCT | 0 | 0 | 0 | 0 | 0 | 0 |
| Polgreen (2015) ^60)^ | RCT | 0 | -1 | 0 | -1 | 0 | 0 |
| Rinfret (2009) ^61)^ | RCT | 0 | -1 | 0 | 0 | 0 | 0 |
| Rohla (2023) ^62)^ | RCT | 0 | -1 | -1 | -1 | 0 | -1 |
| Rudd (2004) ^63)^ | RCT | 0 | -1 | 0 | -1 | 0 | 0 |
| Schwalm (2020) ^64)^ | RCT | 0 | -1 | 0 | -1 | 0 | 0 |
| Shireman (2016) ^65)^ | RCT | 0 | -1 | -1 | 0 | 0 | 0 |
| Svarstad (2013) ^66)^ | CRCT | 0 | -1 | -1 | 0 | 0 | -1 |
| Tobari (2010) ^67)^ | CRCT | 0 | -1 | 0 | 0 | 0 | 0 |
| Towfighi (2021) ^68)^ | RCT | 0 | -1 | -1 | 0 | 0 | 0 |
| Wang (2011) ^69)^ | RCT | 0 | -1 | 0 | -1 | 0 | 0 |
| Wang (2022) ^70)^ | RCT | 0 | -1 | 0 | -1 | 0 | 0 |
| Weber (2010) ^71)^ | RCT | 0 | -1 | 0 | -1 | 0 | 0 |
| Xiao (2020) ^72)^ | RCT | 0 | -1 | 0 | -1 | 0 | 0 |
| Yip (2015) ^73)^ | RCT | 0 | -1 | 0 | -1 | 0 | 0 |
| Zhao (2012) ^74)^ | RCT | 0 | 0 | 0 | 0 | 0 | 0 |
| Zhu (2018) ^75)^ | RCT | -1 | -1 | 0 | -1 | 0 | -1 |
| Abbreviations: CRCT, cluster randomized controlled trial; RCT, randomized controlled trial; R, bias arising from the randomization process; D, bias due to deviations from intended interventions; Mi, bias due to missing outcome data; Me, bias in the measurement of the outcome; S, bias in the selection of the reported result; O, overall risk of bias; 0, low; -1, some concerns | | | | | | | |

**Supplementary Table 4: Effects of team-based care other than CHW-led care for hypertension on the office blood pressure**

| Outcomes | Trials | Mean difference | Heterogeneity (%) | | Test for overall effect | |
| --- | --- | --- | --- | --- | --- | --- |
|  | N | Mean (95%CI) | I^2^ | P value | Z | P value |
| OSBP (mmHg)  ODBP (mmHg) | 51  49 | −5.71 (−6.61, −4.80)  −2.63 (−3.17, −2.09) | 75  69 | <0.001  <0.001 | 12.35  9.56 | <0.001  <0.001 |
| Abbreviations: CHW: community health worker; CI: confidence interval; N: number; ODBP: office diastolic blood pressure; OSBP: office systolic blood pressure | | | | | | |

**Supplementary Table 5: Effects of team-based care other than CHW-led care for hypertension on office systolic blood pressure in subgroups**

| Subgroups | Trials | Mean difference (mmHg) | Heterogeneity (%) | | Test for overall effect | | P heterogeneity |
| --- | --- | --- | --- | --- | --- | --- | --- |
|  | N | Mean (95%CI) | I^2^ | P value | Z | P value | P value |
| Intervention  Pharmacist-led  Nurse-led  Physician-led  Others  Follow-up periods  ≤ 6 months  6 < to ≤ 12 months  > 12 months | 22  16  6  7  32  15  4 | −5.74 (−7.10, −4.38)  −5.79 (−7.69, −3.88)  −4.07 (−7.31, −0.83)  −8.03 (−10.95, −5.10)  −6.18 (−7.29, −5.07)  −4.98 (−7.06, −2.89)  −6.90 (−12.09, −1.71) | 74  74  76  42  60  85  82 | <0.001  <0.001  <0.001  0.11  <0.001  <0.001  <0.001 | 8.27  5.96  2.46  5.37  10.90  4.67  2.60 | <0.001  <0.001  <0.001  <0.001  <0.001  <0.001  0.009 | 0.34  0.57 |
| Abbreviations: CHW: community health worker; CI: confidence interval; N: number | | | | | | | |


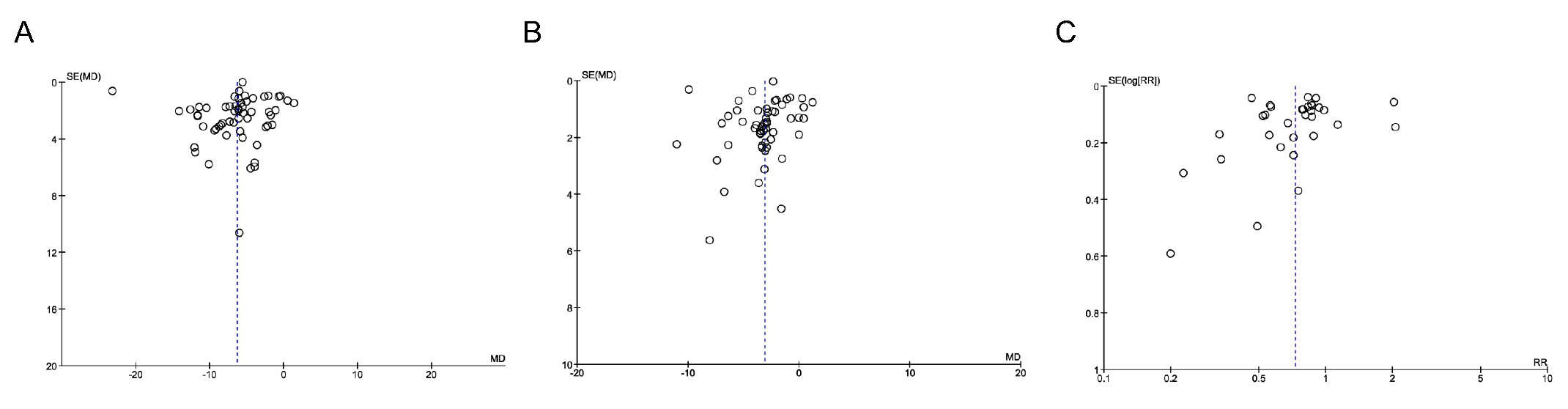


# **Supplementary Figure 1: Funnel plots of effects of patient care team for hypertension on the office blood pressure, and risk for uncontrolled blood pressure**

A: office systolic blood pressure; B: office diastolic blood pressure; C: risk of uncontrolled blood pressure

Abbreviations: CI: confidence interval; MD: mean difference; RR: risk ratio; SE: standard error.


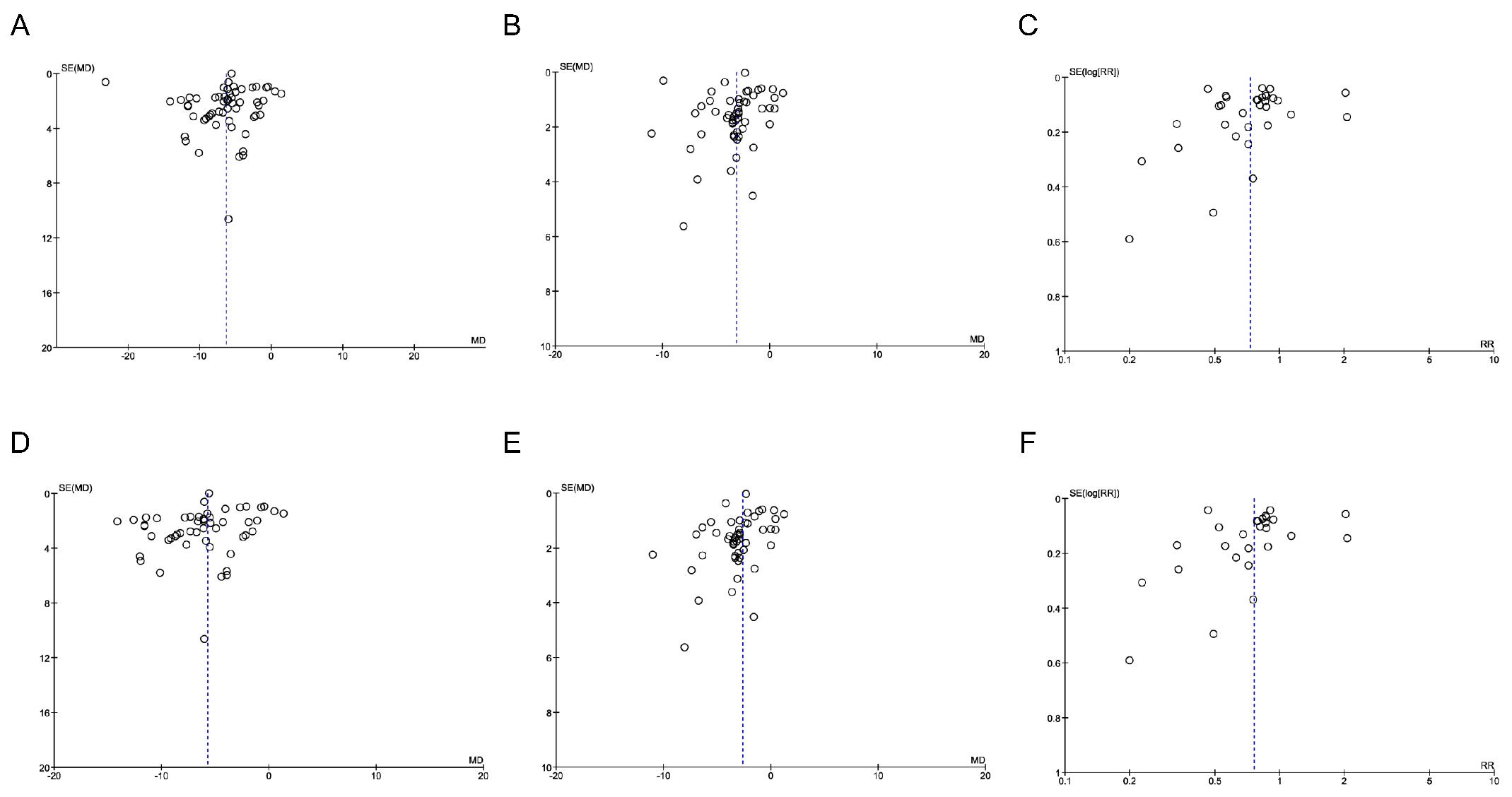


**Supplementary Figure 2: Funnel plots of effects of the patient care team** **other than CHW-led care for hypertension on the office blood pressure, and risk for uncontrolled blood pressure**

D: office systolic blood pressure; E: office diastolic blood pressure; F: risk of uncontrolled blood pressure

Abbreviations: CHW: community health worker; CI: confidence interval; MD: mean difference; RR: risk ratio; SE: standard error.

#
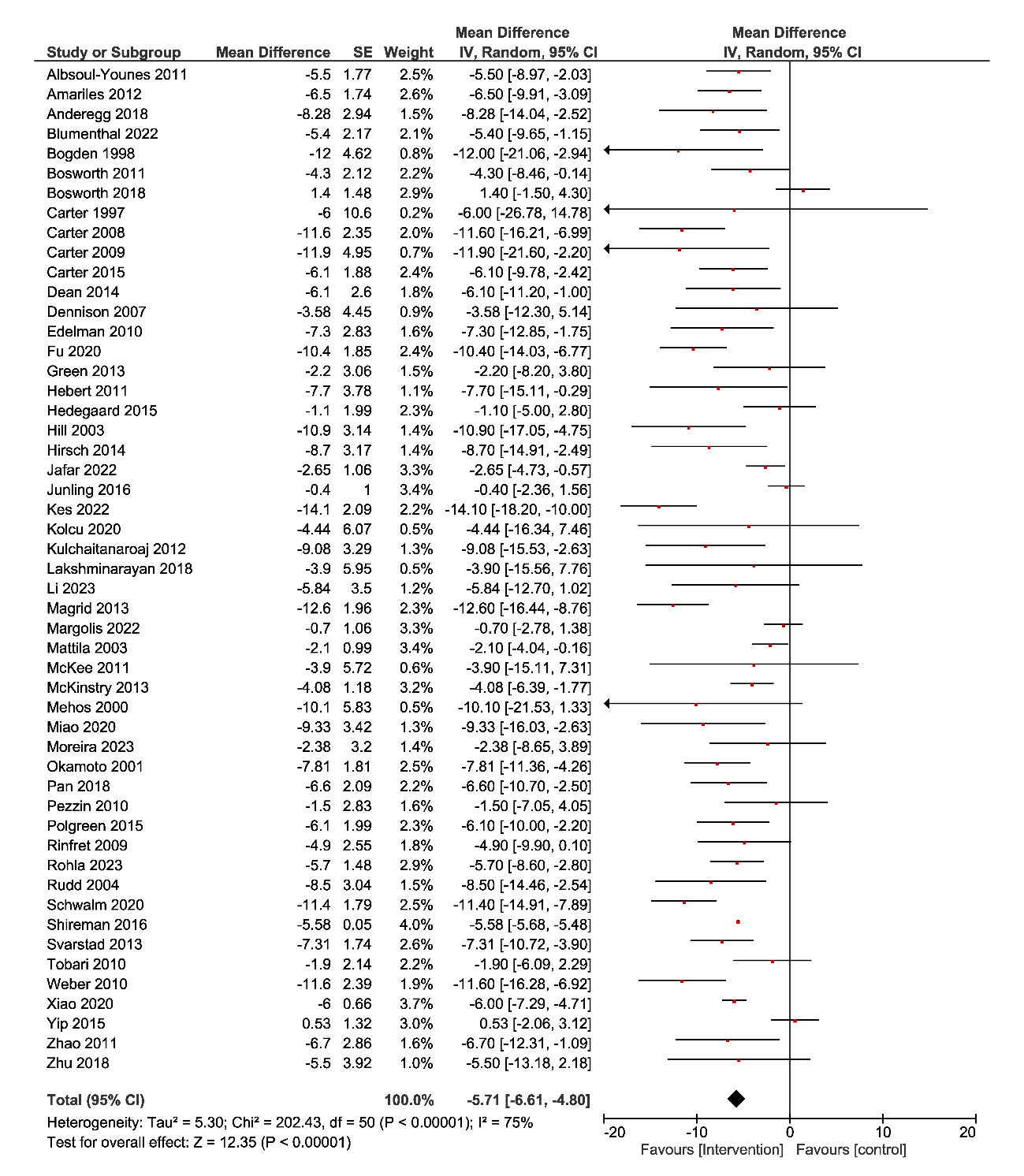


**Supplementary Figure 3**: Effects of the patient care team other than CHW-led care for hypertension on the office systolic blood pressure


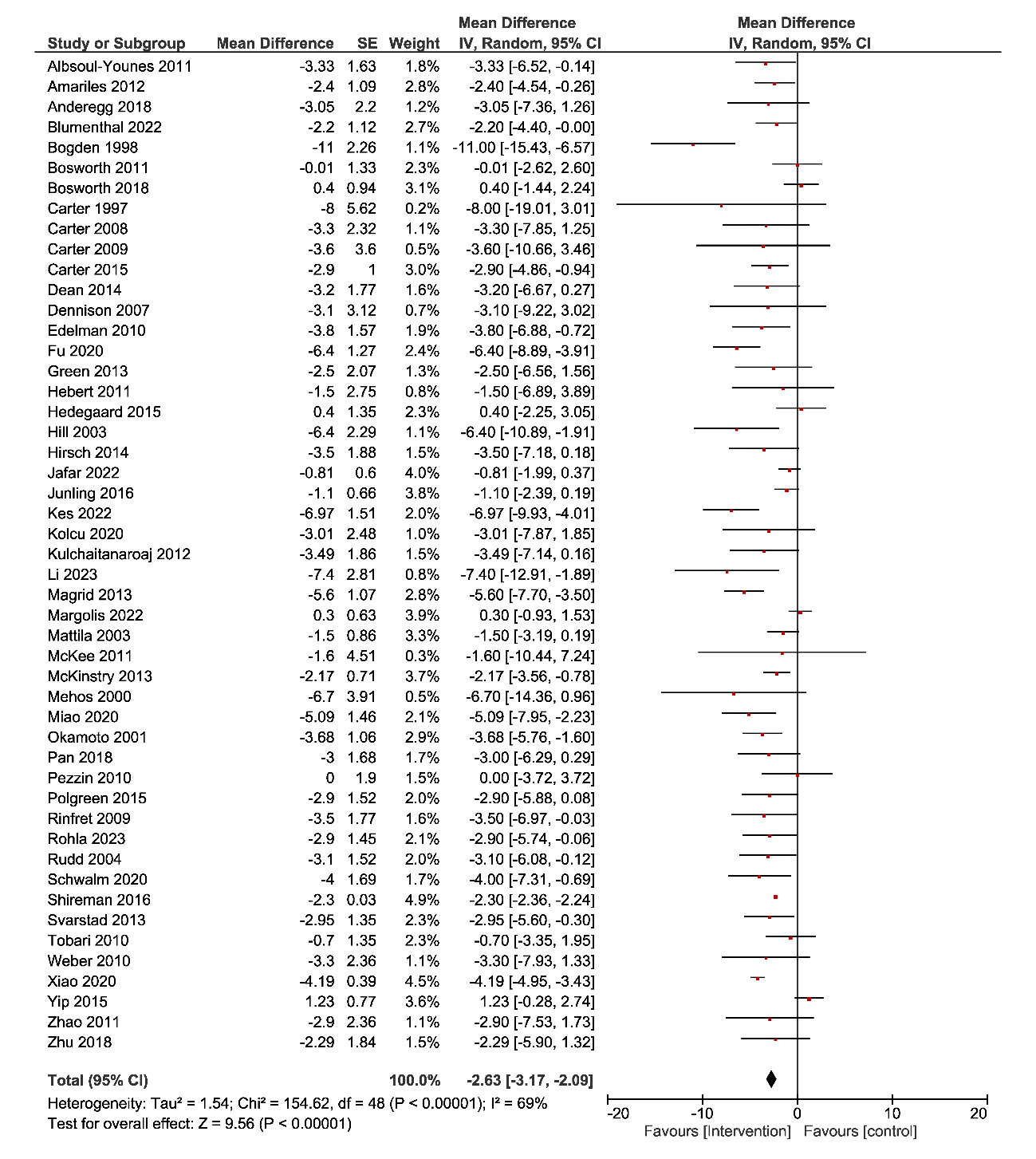


**Supplementary Figure 4**: Effects of team-based care other than CHW-led care for hypertension on the office diastolic blood pressure


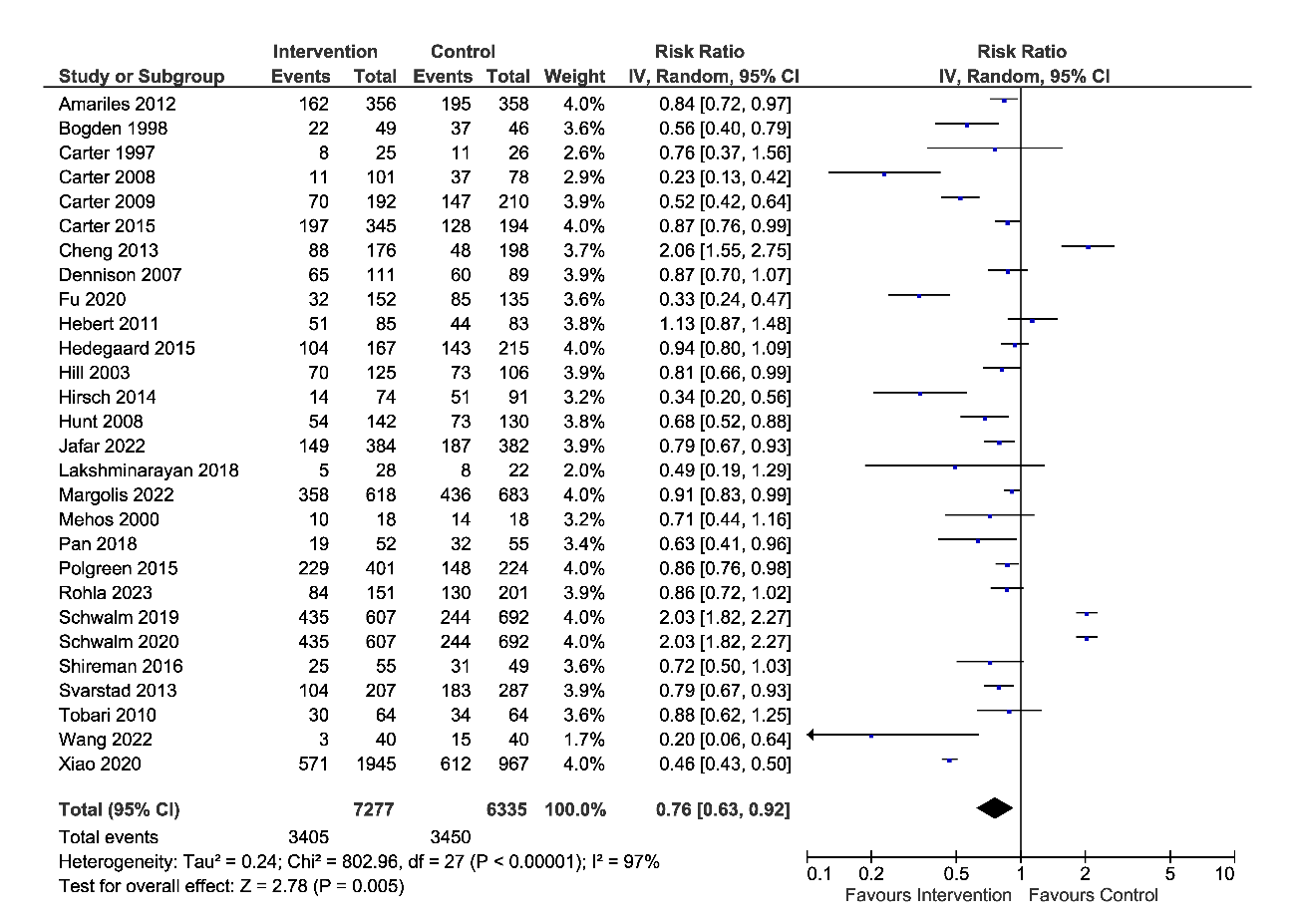


**Supplementary Figure 5**: Effects of team-based care other than CHW-led care for hypertension on the risk for uncontrolled blood pressure

# **Supplementary Text 1: Search strategy**

PubMed Database (Search date: May 17, 2024)

| No | Search terms | n |
| --- | --- | --- |
| #1 | "Patient Care Team"[MeSH Terms] | 73373 |
| #2 | "Practice Patterns, Nurses'"[MeSH Terms] | 2974 |
| #3 | "Practice Patterns, Pharmacists'"[MeSH Terms] | 30 |
| #4 | "Practice Patterns, Physicians'"[MeSH Terms] | 67685 |
| #5 | "Community Health Workers"[MeSH Terms] | 6820 |
| #6 | "team based care"[Title/Abstract] | 1394 |
| #7 | physician*[Title/Abstract] | 478940 |
| #8 | pharmacist*[Title/Abstract] | 45674 |
| #9 | nurse*[Title/Abstract] | 336362 |
| #10 | #1 OR #2 OR #3 OR #4 OR #5 OR #6 OR #7 OR #8 OR #9 | 924582 |
| #11 | "Blood Pressure"[MeSH Terms] | 312557 |
| #12 | Hypertension[MeSH Terms] | 324488 |
| #13 | #11 OR #12 | 547444 |
| #14 | Randomized Controlled Trials as Topic[MeSH Terms] | 173318 |
| #15 | random*[Title/Abstract] | 1519758 |
| #16 | #14 OR #15 | 1579552 |
| #17 | #10 AND #13 AND #16 | 2150 |
| #18 | animals[MH] NOT humans[MH] | 5220572 |
| #19 | #17 NOT #18 | 2146 |
| #20 | Review[Publication Type] | 3326764 |
| #21 | "Systematic Review"[Publication Type] | 260222 |
| #22 | "Meta-Analysis"[Publication Type] | 200239 |
| #23 | "Clinical Trial Protocol"[Publication Type] | 13062 |
| #24 | #20 OR #21 OR #22 OR #23 | 3524175 |
| #25 | #19 NOT #24 | 1803 |

# **Supplementary Text 2: Search strategy**

Cochrane Central Database (Search date: May 17, 2024)

| No | Search terms | n |
| --- | --- | --- |
| #1 | MeSH descriptor: [Patient Care Team] explode all trees | 4,268 |
| #2 | MeSH descriptor: [Practice Patterns, Nurses'] explode all trees | 12,863 |
| #3 | MeSH descriptor: [Practice Patterns, Pharmacists'] explode all trees | 6,263 |
| #4 | MeSH descriptor: [Practice Patterns, Physicians'] explode all trees | 30 |
| #5 | MeSH descriptor: [Community Health Workers] explode all trees | 1,504 |
| #6 | (team based care):ti,ab,kw | 3,675 |
| #7 | (physician*):ti,ab,kw | 3,775 |
| #8 | (pharmacist*):ti,ab,kw | 7,439 |
| #9 | (nurse*):ti,ab,kw | 12,079 |
| #10 | #1 OR #2 OR #3 OR #4 OR #5 OR #6 OR #7 OR #8 OR #9 | 31,879 |
| #11 | MeSH descriptor: [Blood Pressure] explode all trees | 70,907 |
| #12 | MeSH descriptor: [Hypertension] explode all trees | 84,418 |
| #13 | #11 OR #12 | 210 |
| #14 | #10 AND #13 | 1,312,476 |
| #15 | MeSH descriptor: [Randomized Controlled Trials as Topic] explode all trees | 1,334,916 |
| #16 | (randomiz*):ti,ab,kw | 2,966 |
| #17 | (randomis*):ti,ab,kw | 1,332,090 |
| #18 | (randomly):ti,ab,kw | 33,918 |
| #19 | #15 OR #16 OR #17 OR #18 | 26,563 |
| #20 | #14 AND #19 | 111,552 |
| #21 | [mh animals] NOT [mh humans] | 121,055 |
| #22 | #20 NOT #21 | 6,0352 |
| #23 | MeSH descriptor: [Review Literature as Topic] explode all trees | 5,174 |
| #24 | MeSH descriptor: [Systematic Reviews as Topic] explode all trees | 4,487 |
| #25 | MeSH descriptor: [Meta-Analysis as Topic] explode all trees | 4,366 |
| #26 | MeSH descriptor: [Clinical Trial Protocols as Topic] explode all trees |  |
| #27 | #23 OR #24 OR #25 OR #26 |  |
| #28 | #22 NOT #27 |  |

# **Supplementary Text 3: Search strategy**

IchuShi Web Database (Search date: May 22, 2024)

| No | Search terms | n |
| --- | --- | --- |
| #1 | (チーム医療/TH or チーム医療/AL or "Patient Care Team"/AL ) | 132716 |
| #2 | (看護師/TH or 看護師/AL or nurse/AL) | 183288 |
| #3 | (薬剤師/TH or 薬剤師/AL or pharmacist/AL) | 72068 |
| #4 | (医師/TH or 医師/AL or physician/AL) | 448986 |
| #5 | "(保健医療従事者/TH or 医療従事者/AL) or |  |
| #6 | (""コミュニティヘルスワーカー""/TH or ""community health worker""/AL)" | 960489 |
| #7 | #1 OR #2 OR #3 OR #4 OR #5 | 10711 |
| #8 | ((血圧/TH or 血圧/AL or "blood pressure"/AL)) and (PT=原著論文, 議録除く) | 115303 |
| #9 | ((高血圧/TH or 高血圧/AL or "hypertension"/AL)) and (PT=原著論文, 会議録除く) | 159842 |
| #10 | #7 OR #8 | 56212 |
| #11 | ((ランダム化比較試験/TH or ランダム化比較試験/AL or RCT/AL or "randomized controlled trial"/AL or "randomised controlled trial"/AL)) and (PT=原著論文, 会議録除く) | 158 |
| #12 | #6 AND #9 AND #10 | 21184 |
| #13 | レビュー論文/AL or Review/AL and (PT=原著論文, 会議録除く) | 4689 |
| #14 | (システマティックレビュー/TH or システマティックレビュー/AL) or ("Systematic Review"/AL) and (PT=原著論文, 会議録除く) | 8927 |
| #15 | (メタアナリシス/TH or メタアナリシス/AL) or メタアナライシス/AL or "Meta-Analysis" and (PT=原著論文, 会議録除く) | 9311 |
| #16 | (臨床試験プロトコール/TH or プロトコール/AL) or "Clinical Trial Protocol"/AL and (PT=原著論文, 会議録除く) | 39074 |
| #17 | #12 OR #13 OR #14 OR #15 | 147 |
